# College of Physicians of Philadelphia

**Published:** 1842-03-12

**Authors:** 


### PROCEEDINGS OF SOCIETIES.

### COLLEGE OF PHYSICIANS OF PHILADELPHIA.

*Session of December 7th*, 1841. Dr. Fox in the Chair.

This session was principally occupied by Dr. Isaac Parrish, who offered some remarks upon a singular permanent change of voice, occasionally produced by the excision of the tonsils. It was described as "a peculiar, shrill, nasal twang in pronouncing certain words," so modifying the speech of the patient as to be exceedingly unpleasant. Dr. P. had met with two cases of this character, and deemed the subject one of grave importance, in deciding upon the propriety of operating in chronic enlargement of the tonsils. He appeared inclined to attribute the evil under notice, to injury done the lateral half-arches of the palate by the operation in cases of the adhesions between them and the enlarged tonsils, which are so commonly observed after the acute form of cynanche tonsillaris.

"Since my attention has been directed to this subject, I have in several instances observed adhesions to exist between enlarged tonsils and the edge of the soft palate, before any operation had been performed. In these cases the patients had suffered from attacks of acute tonsillitis, of which these adhesions were probably the result. Would not the extirpation of a tonsil thus confined, be followed by the condition of parts here pointed out, unless the adhesions were first liberated? In one instance, I was deterred from the operation for fear of this result. And should it be necessary to perform it, the probability of a change of voice after the operation ought to be taken into account. So far as I know, the attention of surgeons has not been directed to this peculiar pathological condition, and to its effect upon the function of speech.

"The subject is certainly deserving of attention, and is now introduced to the College in the hope of eliciting further information. The part which the soft palate plays in modulating the voice, is perhaps not accurately determined; it is well known, however, that its integrity, and freedom of motion in its muscles, are necessary to the perfection of this important function. In a recent elaborate memoir upon the mechanism of the voice presented to the French Academy of Medicine by Dr. Bennati, physician to the Italian Theatre of Paris, and himself a musician, it is asserted, that the soft parts of the larynx exert a most important influence in modulating sound. And he has investigated with considerable minuteness the effect of certain diseases of these parts, in modifying the notes of speech, more particularly in singers. It is well known, and the observations of Beunate abundantly confirm the fact, that aphony, either partial or complete, is frequently produced by a pathological condition of the mucous membrane of the soft palate and pharynx without any obvious diseases of the larynx, or the parts more immediately concerned in the production of sound. Thus even an elongation of the uvula, accompanied as it generally is with a relaxed condition of the palate, is known to affect the strength of the voice very materially, and even sometimes to prevent its prolonged exercise in public speaking or singing."

Drs. Bond, Condie, and B. H. Coates made a few inquiries of the author of the paper, but neither of them appears to have met with changes of voice of the character described.

*Session of Jan. 4th,* 1842. Dr. Morris in the Chair.

Dr. Morris read the following account of "*a case in which death resulted from an abscess behind the pharynx*," noticed by him verbally at the meeting of October last.

Mrs. P., of delicate constitution, slight person, and pallid complexion, aged 25 years, about the middle of the ninth month of utero gestation, consulted me on the afternoon of Thursday, the 7th of May, complaining of a slight degree of soreness of the right side of the neck, about half way between the angle of the jaw and the clavicle. There was, when I saw her, some fever, which had been preceded by a slight chill. On Tuesday she had fatigued herself by an unusually long walk, and on Wednesday had ridden into the country, and spent a considerable time on the damp ground, walking and standing. There was no external swelling, nor was there any redness or other indication of inflammation of the tonsils or pharynx.

A smart saline purgatixe subdued the fever, and relieved her so much, that on Saturday she was able to attend to her domestic duties, and spent the evening with much enjoyment in the family circle. A severe chill on Saturday morning ushered in a renewed attack. The fever was very high. There was a slight degree of swelling and redness of the soft palate and uvula, no external swelling and no enlargment of the tonsils. Bleeding was proposed, but postponed in compliance with the prejudices of the patient. An active saline cathartic was administered, and a common gargle directed. On Monday morning I found she had passed a sleepless night, sitting in the lap of her husband, from her dread of strangulation should she lie down. There was a slight cough, and much greater difficulty of swallowing than could be attributable to the apparent condition of the throat, which was examined with great care, the tongue being depressed, and the mouth well opened. The heat of the skin was very great, but without redness, the circulation was rapid, and the countenance expressive of distress. Bleeding was now insisted on, and practised promptly and largely, without, however, affording any decided relief. Leeches were applied to the throat in the afternoon, and morphia administered at night, much of the restlessness of the preceding night, and of the dysphagia being ascribed to the excitable condition of the nervous system, owing to her approaching accouchement; respecting which she had much anxiety, excited by the death of an intimate friend in childbed a short time before.

Tuesday morning found her with an increase of all the symptoms; she had passed a sleepless night; her pulse was very rapid, soft, and voluminous, the heat of the skin very great. She was unable to raise the natural tones of her voice; the uvula was slightly swollen, and there were some small deposits of lymph upon it. There was a little cough. I bled her again freely, but without any mitigation of her symptoms; she drank with tolerable ease, but was unable to swallow solids, or to lie down. There was no apparent increase of swelling, and nothing visible which could account for the great difficulty in swallowing. The tongue was clean and pallid. The common gargles were used freely, and promoted an abundant discharge of mucus from the fauces. She took, during this day, Tuesday, freely of gruel and some ice cream. Morphia was again resorted to in the hope of procuring sleep at night; and though the symptoms were of so anomalous a character, no apprehensions were excited of an unfavourable result. Wednesday morning found her with entire aphonia, slight cough, and utter inability to swallow; the fever was unabated; the bowels had been freely opened by cathartics given the previous day. Gargles were applied with a syringe, and always with some relief, and frequently she was able to swallow small portions immediately after their use. I examined the fauces and the neck at each visit, but without being able to ascertain any cause for the urgency of the symptoms; a blister was applied to the throat and the gargles were employed as before; and as there was no marked difficulty of breathing or other symptoms indicating urgent danger, an anodyne was given, and she was left for the night. About 11 o'clock labour commenced, and Dr. Hodge was summoned to see her at my request. There appeared at this time an abatement of all the symptoms; she lay quietly on her left side, slept between the pains, which were regular and efficient, and spoke freely with her natural tone of voice. Dr. Hodge retired to rest, and I remained with her during the greater part of the night. Towards morning there appeared to be a cessation of the expulsive effort, although the uterine contractions occurred at the usual intervals, and Dr. H. was called to see her. The heat of the skin was at this time so great, and the pulse so voluminous and rapid, that Dr. H. suggested the propriety of further blood letting; it was not, however, put in practice; but about 7 in the morning of Thursday, the head presenting naturally and at the lower strait, the pains being suspended, the forceps were applied, and she was promptly and safely delivered. The placenta was soon expelled, the uterus contracted firmly, and not more than ten or twelve ounces of blood were lost. The volume and force of the pulse at the wrist was instantly reduced, but its frequency remained undiminished. We remained with her some hours endeavouring to procure the passage of some food into the stomach, but ineffectually. She could sit up, allowed a weak solution of sulphate of copper to be injected into her throat, which she threw again from her mouth, but could not swallow. Her voice was hoarse, but there was little cough, and no difficulty of respiration. I saw her again about 12 o'clock, without finding any change in the case. About two I was summoned to her, and, fearing hæmorrhage, or some urgent symptom, dispatched the messenger who had come for me to call Drs. Hodge and Meigs. We all reached her chamber at nearly the same time, and found her unable to lie down or to swallow, with a very rapid pulse, a warm moist skin, and perfect mental composure. Various efforts were made to ascertain the seat of her difficulty. There was no swelling to account for it, no appearance of disease about the throat internally, nor any cerebral symptoms, and yet our patient was evidently in a condition of great danger; the respiration was unobstructed, though the voice had again sunk to a nearly inarticulate whisper. There was no sound like that of croup in respiration, and no evidence of effusion about the larynx; there was no hæmorrhage nor other cause of exhaustion. Stimulating and nutricious enemata were given freely, and the stomach tube, at the suggestion of Dr. Hodge, was procured, with the intention of supporting her by food injected into the stomach; we were, however, deterred from its use by an apprehension lest it should bring on a sudden spasm of the glottis, and cause immediate death. We all saw her repeatedly from this time until Friday morning, at 11 o'clock, when she expired.

The interesting points in this case are the intensity of the arterial excitement, the dysphagia and aphonia, without a corresponding difficulty of respiration, or sufficient swelling and inflammation in those parts of the throat within sight, and commonly affected, to account for these symptoms. It was not laryngitis, nor bronchitis, nor pharyngitis, nor tonsillitis. The examination of the body revealed the whole mystery. Upon opening the trachea and larynx the traces of inflammation were so slight as hardly to be recognised; and we were disposed at one time to seek the causes of death in the brain, or some other organ. It was, however, determined to remove entirely the pharynx, together with the base of the tongue, in order to look at them carefully from behind; in doing this an abscess was opened, situate between the œsophagus and the vertebra, containing about half an ounce of purulent matter, and so immediately behind the glottis as to account most satisfactorily for the difficulty of swallowing and dread of strangulation expressed by the patient, from the time the disease first assumed a serious character. There were also minute depositions of pus between the arytenoid and cricoid cartilages, shewing the cause of the difficulty of speaking.

*(To be continued.)*

